# Finite element analysis of load transition on sacroiliac joint during bipedal walking

**DOI:** 10.1038/s41598-020-70676-w

**Published:** 2020-08-13

**Authors:** Ryota Toyohara, Daisuke Kurosawa, Niels Hammer, Michael Werner, Keita Honda, Yusuke Sekiguchi, Shin-Ichi Izumi, Eiichi Murakami, Hiroshi Ozawa, Toshiro Ohashi

**Affiliations:** 1 Division of Human Mechanical Systems and Design, Graduate School of Engineering, Hokkaido University, Kita 13, Nishi 8, Kita-ku, Sapporo, Hokkaido 060-8628 Japan; 2 Department of Orthopedic Surgery/Low Back Pain and Sacroiliac Joint Center, JCHO Sendai Hospital, Sendai, Japan; 3 Department of Clinical and Macroscopic Anatomy, Medical University of Graz, Graz, Austria; 4 Department of Orthopedic and Trauma Surgery, University of Leipzig, Leipzig, Germany; 5 Fraunhofer IWU, Medical Branch, Dresden, Germany; 6 Institute of Anatomy, University of Leipzig, Leipzig, Germany; 7 Department of Physical Medicine and Rehabilitation, Tohoku University Graduate School of Medicine, Sendai, Japan; 8 Graduate School of Biomedical Engineering, Tohoku University, Sendai, Japan; 9 Department of Orthopaedic Surgery, Faculty of Medicine, Tohoku Medical and Pharmaceutical University, Sendai, Japan; 10 Division of Mechanical and Aerospace Engineering, Faculty of Engineering, Hokkaido University, Sapporo, Japan

**Keywords:** Musculoskeletal models, Computer modelling, Musculoskeletal system

## Abstract

The sacroiliac joint (SIJ) is burdened with variant loads. However, no methods have allowed to measure objectively how the SIJ deforms during bipedal walking. In this study, in-vivo walking conditions were replicated in a kinematic model combining the finite element method with 3D walking analysis data divided into five phases in order to visualize the load transition on the SIJ and clarify the role of the SIJ. Both models with and without inclusion of the SIJ were investigated. In models with bilateral SIJs, the displacement differed greatly between the sacrum and both hip bones on the SIJ as the boundary. The movements of the sacrum involved a nutation movement in the stance phase and a counter-nutation in the swing phase relative to the ilium. In models without SIJs, the displacement of the pelvis and loads of pelvic ligaments decreased, and the equivalent stress of the SIJs increased compared to the model with SIJs. The walking loads cause distortion of the entire pelvis, and stress concentration at the SIJ are seen due to the morphology of the pelvic ring. However, the SIJs help dissipate the resulting stresses, and the surrounding ligaments are likewise involved in load transmission.

## Introduction

The sacroiliac joint (SIJ) forms the junction between the sacrum and the ilium, and is composed of synovial joints in the anterior third and tough ligaments in the posterior aspect^1^. Since these strong ligaments support the SIJ, it has comparably low mobility. The range of its motion is generally considered to be a few millimeters and degrees^2–5^. The SIJ is largely assumed to serve as a damper, receiving impact between the upper and lower part of the body, thereby transmitting effectively. However, the joint line of the SIJ runs roughly parallel to the line gravity, i.e., shear force is generated at the SIJ from an upper body weight^6^. One of the representative movements of the SIJ is nutation and counter-nutation, which is forward and backward rotation of the sacrum on sagittal plane, respectively. The sacrum is in a standing state, and the wedge is deeply driven into the pelvic ring fixed by the pubic symphysis and the posterior ligaments. This is considered to be the strongest state (close-packed position)^7^. Pain arising from the SIJ is considered to occur due to an unexpected force or repeated impact^8^. There are several treatment options to relieve SIJ pain such as physical therapies, pelvic orthotics and the injection of local anesthetics as conservative treatment, as well as SIJ fusions using implants as surgical treatments. Since SIJ dysfunction is hypothesized to be caused by joint misalignment, fixing the SIJ and preventing excessive motion is considered effective in relieving the pain.

Bipedal walking consists of a period where only one leg supports the total body weight in the walking cycle. In this period, shear forces are stronger compared to standing on both legs in a resting state. During the swing phase, the SIJ is pulled downward by the weight of the free leg. Therefore, the SIJ is burdened with variant loads during walking. However, no methods could to date measure objectively how the SIJ deforms in vivo as well as in vitro during the different phases of walking. Previous research made attempt to quantify the SIJ motion by three-dimensional computed tomography (3D-CT) and by loading tests on cadavers, reporting that the SIJ moves less than 1 mm and does function as a joint^5^. These trials were however conducting under static conditions.

In this study, computer simulations were performed using finite element analysis during normal walking, resembling the five walking phases of a living body. The finite element model of the pelvis used in this experiment has reproduced the SIJ and the surrounding ligaments by previous anatomical studies^9–13^. In addition, this finite element model included both femora, and simulations can be performed with walking data.

The aim of the present study was to determine the dynamic load transition on the SIJ and to clarify the role of the SIJ. It was hypothesized that (A) the stress increases in the pelvis if the SIJ is lost, and (B) the SIJ plays an important role during walking.

## Methods

### Finite element model

**Figure 1:**
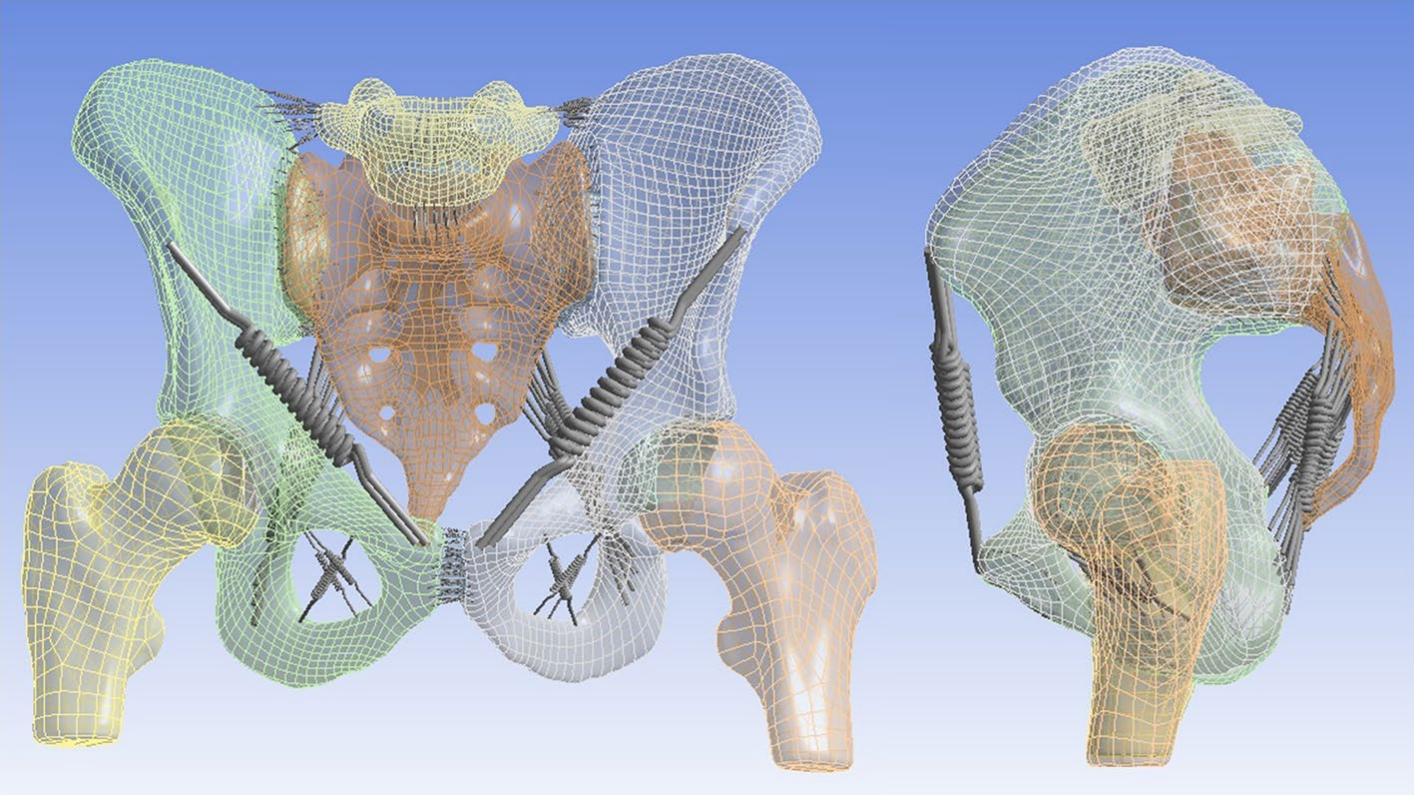
Anterior view (left) and left lateral view (right) of the pelvic model used for finite elements simulations.

**Figure 2:**
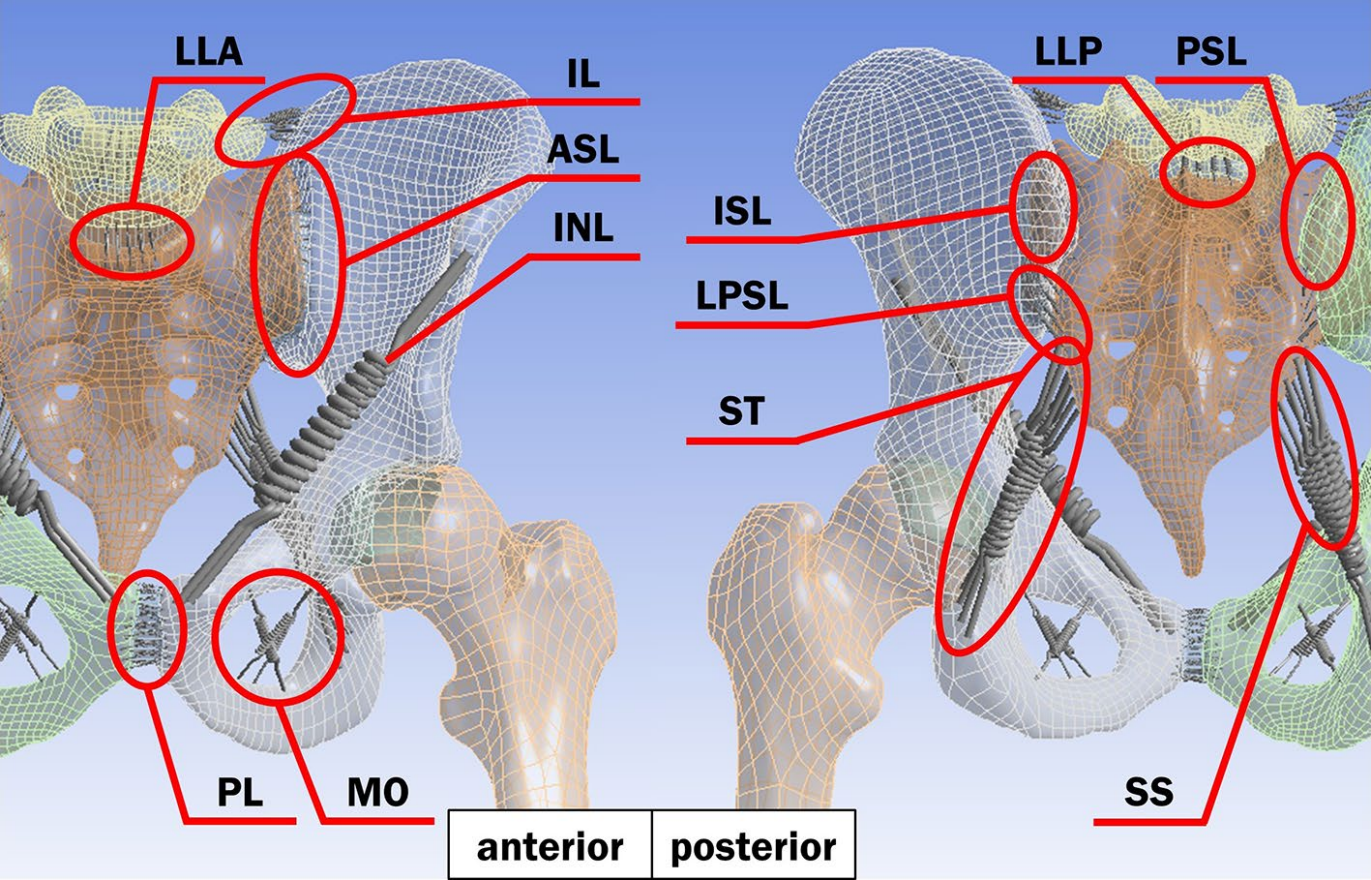
The positions and the names of ligaments in the pelvic model with anterior (left) and posterior (right) views. The ligaments are iliolumbar ligament (IL), anterior longitudinal ligament (LLA), posterior longitudinal ligament (LLP), anterior sacroiliac ligament (ASL), interosseous sacroiliac ligament (ISL), posterior sacroiliac ligament (PSL), long posterior sacroiliac ligament (LPSL), inguinal ligament (INL), sacrospinous ligament (SS), sacrotuberous ligament (ST), pubic ligament (PL) and obturator membrane (MO).

The finite element model of the pelvis (Fig. 1) was created based on CT of a healthy male pelvis (29 years old, 185 cm, 69 kg), and imported into ANSYS 19.2 (Cybernet Systems Co., Ltd., Tokyo, Japan)^14^. This FE model included the fifth lumbar vertebra, the sacrum, both hip bones and proximal ends of both femora, as well as both SIJs cartilage, the pubic symphysis, both hip joints cartilage and the intervertebral disks (Fig. 1). A total of 210 spring elements representing the ligaments surrounding the pelvis were modelled, considering the major fiber orientations and based on previous anatomical studies on more than 80 cadavers^9,10,13^. 12 types of ligaments were modelled (Fig. 2). The ligaments were defined in a way where they act only when they are subjected to tensile loads because they do not have a stabilization property on the pelvis and cannot sustain the bones in the case of a compressive load.

### Material properties

All tissues were defined as a uniform isotropic material for simplification and their material properties were referred from the paper by Wirtz et al.^15^ and experimental results by Hammer et al. (Table 1). The hyperelastic material properties based on Mooney-Rivlin model, which is the strain energy density function W given by following formula, as a complete non-compressional body.

$$\mathrm{W}={C}_{10}\left({I}_{1}-3\right)+{C}_{01}\left({I}_{2}-3\right)$$

Here, ${C}_{10}$ and ${C}_{01}$ are material constants, and ${I}_{1}$ and ${I}_{2}$ are the first and second invariant of the distortion.

**Table 1:** Material properties. C10, C01 and C11 mean the parameters of Mooney–Rivlin model for hyperelastic bodies.

Material	Tissue	Young’s modulus (MPa)	Poisson’s ratio	C10 (MPa)	C01 (MPa)	C11 (MPa)
Cortical bone	Both hip bones, Sacrum	11,000	0.2	–	–	–
	Both femora, Both SIJ cartilage					
	5th lumbar vertebra					
Cartilage (elastic body)	Both hip joints cartilage	150	0.2	–	–	–
	intervertebral disks					
SIJ cartilage (hyperelastic body)	Both SIJs cartilage	–	–	4.1	0.41	0
Symphysis cartilage (hyperelastic body)	Pubic symphysis	–	–	0.1	0.45	0.6
Ligament	All ligaments	350	–	–	–	–

### Mesh Generation

The bones and joints were meshed using tetrahedral elements consisting of 10 nodes each. The total number of elements and nodes for this model was 141,672 and 80,578, respectively. The average element quality is 0.75, indicating good mesh quality. This is a composite quality metric given in the ranges between 0 and 1, and a value of 0 and 1 indicates the element has a zero volume and a perfect cube, respectively.

### Loading and boundary conditions

**Figure 3:**
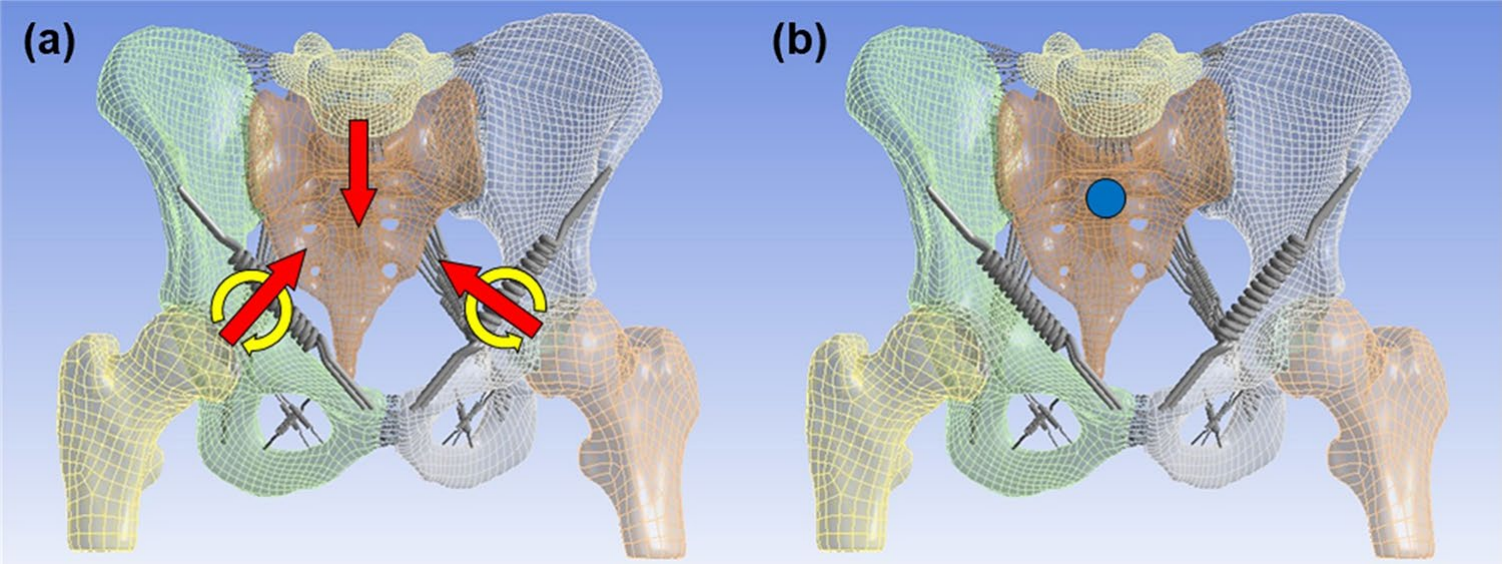
(**a**) The yellow arrows indicate where the joint moments are applied, the red arrows indicate where the surface loads are applied, and (**b**) the blue point indicates where the pelvis is fixed.

Walking parameters (Table 2) were obtained with 3D walking analysis (MAC 3D, Motion Analysis Corporation, Rohnert Park, CA, USA) of six healthy people (4 males, 2 females, an average of 26.7 years old) by Department of Physical Medicine and Rehabilitation, Tohoku University Graduate School of Medicine (Tohoku University hospital ethics committee, Approval No. 2018-1-552)^16^. All methods were performed in accordance with relevant guidelines and regulations, and all participants provided written informed consent before participating in the experiment. Five walking phases were defined, focusing on the right leg and being performed in a static simulation environment, and were named phases 1 to 5, respectively. In order to simulate walking conditions properly, the joint moments were applied on both femoral heads and surface loads were applied on adhesive surfaces of both femoral heads and both hip bones cartilage and on the base of the sacrum (Fig. 3a). The surface loads on the base of the sacrum were calculated from the ones on both femoral heads with the principal of action and reaction. By changing the angle of joint moments and surface loads, the tilt, drop and rotation of the pelvis were reproduced during walking. The anterior aspect of the second sacral spine was fixed in space in order to reproduce movements of the sacrum: nutation and counter-nutation (Fig. 3b). For contact type, all surfaces in contact were defined as “bonded”, which means the surfaces are fixed to each other.

**Table 2:** Mean values of joint moments, surface loads, and pelvic angles.

			Phase 1	Phase 2	Phase 3	Phase 4	Phase 5
Joint moments^1^ (Nm)	Right femur	Sagittal plane	10.9 (19.9, 1.8)	− 18.9 (− 16, − 21.2)	− 21.3 (− 17.4, − 29.1)	− 2.5 (− 1.2, − 3.9)	0.5 (1.6, − 0.5)
		Coronal plane	17.8 (23.1, 11.9)	44.5 (50.1, 35.9)	16.5 (22.8, 13.9)	0.5 (0.8, 0.2)	0.1 (0.2, − 0.1)
		Transversal plane	5.8 (9.2, 3.8)	0 (1.4, − 1)	9.5 (15.9, 5.7)	− 0.2 (− 0.1, − 0.3)	0.1 (0.4, 0)
	Left femur	Sagittal plane	− 17 (− 13.2, − 24)	− 0.8 (− 0.5, − 1.1)	16.1 (21.3, 9.4)	− 5.7 (1.8, − 12.8)	− 28.1 (− 20.6, − 41)
		Coronal plane	16.4 (20.7, 11)	0.3 (0.5, 0.2)	15 (21.5, 10.9)	45 (51.6, 36.3)	41.6 (52.3, 32.7)
		Transversal plane	10.2 (18, 3.8)	0 (0, − 0.1)	− 6.4 (− 1.9, − 11.6)	− 5.1 (− 1.7, − 9.9)	3.2 (5.5, 0.4)
Surface loads^2^ (N)	Right femur	Medio-lateral	13.9 (21.3, 6.5)	36.1 (48.9, 25.4)	16.7 (29, 8.9)	1.7 (2.4, 1.1)	1.7 (2.6, 1)
		Anterior–posterior	53 (65.6, 40.9)	− 0.8 (3.6, − 6.7)	− 74 (− 62.7, − 101.7)	− 3.9 (− 2.7, − 5.1)	2.6 (3.3, 1.7)
		Vertical	344.6 (422, 244.5)	558.3 (659.1, 438)	319 (424.8, 255.6)	− 26.5 (− 21.2, − 31.2)	− 25.6 (− 20.8, − 30.1)
	Left femur	Medio-lateral	− 18 (− 10.3, − 26.3)	− 1.7 (− 1.3, − 2.2)	− 11 (− 6.3, − 17.6)	− 33.7 (− 24.6, − 45.1)	− 34.3 (− 22.1, − 45.3)
		Anterior–posterior	− 66.9 (− 57.6, − 82)	− 0.4 (0.2, − 1)	46.8 (66.9, 28.1)	40.4 (55.8, 30.7)	− 31.3 (− 25.4, − 48.7)
		Vertical	289 (368.8, 235.7)	− 25.8 (− 20.5, − 30.1)	318.6 (403.9, 237)	550.5 (657.5, 424)	553.8 (648.5, 428.5)
	Base of sacrum	Medio-lateral	4.1 (8.2, 0.8)	− 34.4 (− 23.8, − 46.9)	− 5.7 (− 1.9, − 12.1)	32 (42.7, 23.5)	32.6 (43.6, 21.1)
		Anterior–posterior	13.9 (20.4, 0.3)	1.2 (7.3, − 3.3)	27.2 (45.9, 14.6)	− 36.5 (− 28.1, − 51)	28.7 (45.4, 22.9)
		Vertical	− 633.6 (− 480.2, − 760.4)	− 532.5 (− 417.5, − 629.1)	− 637.6 (− 492.6, − 773.1)	− 524 (− 402.8, − 626.3)	− 528.2 (− 407.5, − 619.7)
Pelvic angles^3^ (°)		Sagittal plane	8.1 (12, 3.8)	8.1 (11.2, 5.3)	8.3 (11.5, 4.6)	7.7 (11, 3.8)	7.8 (10.9, 4.6)
		Coronal plane	− 3.8 (− 1.9, − 5.9)	− 1.7 (0.6, − 4.1)	1.6 (3.1, − 0.4)	1.3 (3.8, − 0.5)	− 2.3 (− 0.1, − 3.9)
		Transversal plane	− 5 (− 1.8, − 9)	− 0.8 (0.9, − 3)	3.4 (7.6, − 1.2)	2.6 (7.1, − 1.2)	− 3.1 (− 0.6, − 5.4)

^1^Sagittal plane: flextion (+)/extension (−), coronal plane: abduction (+)/adduction (−), transversal plane: external rotation (+)/internal rotation (−).

^2^Medio-lateral: left (+)/right (−), anterior–posterior: posterior (+)/anterior (−), vertical: superior (+)/inferior (−).

^3^Sagittal plane: anterior tilt (+)/posterior tilt (−), coronal plane: elevation (+)/depression (−) on right leg, transversal plane: forward rotation (+)/backward rotation (−) on right leg.

### Measured parameters

In this study, the resultant displacement of the pelvis and SIJ cartilage, and the equivalent stress (Von Mises Stress) of the SIJ cartilage were investigated. The resultant displacement shows a 3D displacement value and direction. The equivalent stress is a scalar value that is calculated from normal stresses and shear stresses without any distinction between tension and compression. This stress is given by following formula.

$${\sigma }_{eqv}=\sqrt{\frac{1}{2}\{{\left({\sigma }_{xx}-{\sigma }_{yy}\right)}^{2}+{\left({\sigma }_{yy}-{\sigma }_{zz}\right)}^{2}+{\left({\sigma }_{zz}-{\sigma }_{xx}\right)}^{2}+6({\sigma }_{xy}^{2}+{\sigma }_{yz}^{2}+{\sigma }_{xz}^{2})\}}$$

Here, ${\sigma }_{eqv}$ are Von Mises stress, and ${\sigma }_{xx}$, ${\sigma }_{yy}$ and ${\sigma }_{zz}$ normal stress in X, Y and Z direction, respectively. ${\sigma }_{xy}$, ${\sigma }_{yz}$ and ${\sigma }_{xz}$ mean shear stress in XY, YZ and XZ direction, respectively.

In addition, the maximum elastic force of spring probes was investigated for loads on ligaments and summed for each of the ligaments.

### Analytical model

In order to determine the role of the SIJ in the pelvis, both models with and without the SIJ cartilage were created by changing the material property of both SIJ cartilage, and were named “cartilage model” and “bone model”, respectively. The material property used for both SIJ cartilage was the one of the SIJ cartilage (hyperelastic body) in the cartilage model and the one of the cortical bone in the bone model.

## Results

The data obtained from 3D walking analysis yielded consistent results, consequently, one of the datasets of a 33-year-old male (175 cm, 72 kg) was used for the modeling.

### Displacement distribution within pelvis

**Figure 4:**
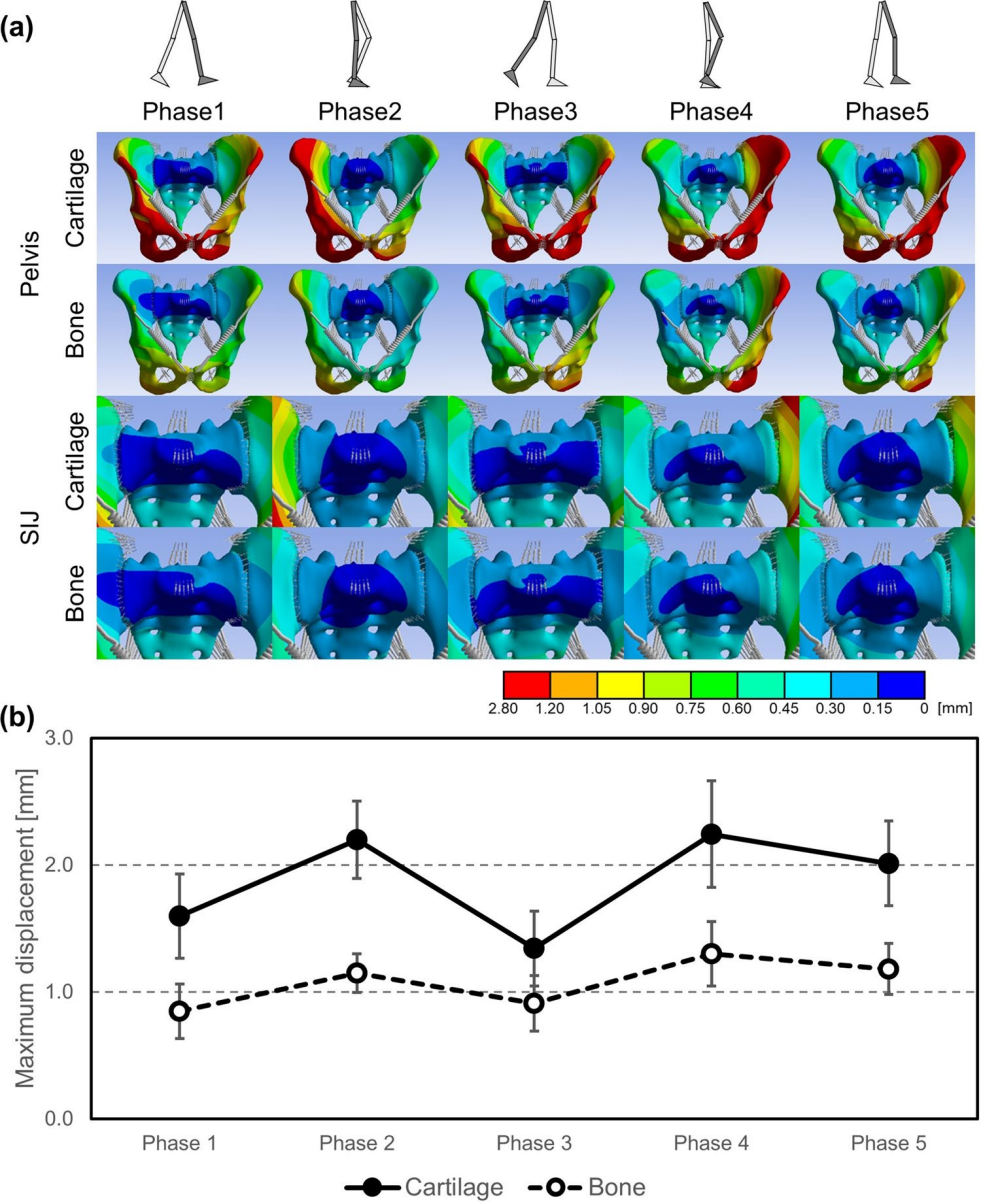
(**a**) Resultant displacement distribution of pelvis excluding femora (top 2 lines) and pelvis surrounding sacroiliac joints (bottom 2 lines) on a representative example, and (**b**) change of mean values of maximum resultant displacement in pelvis excluding femora.

The difference in displacement between the sacrum and ilium in all walking phases averaged 0.23 mm in the cartilage model and 0.04 mm in the bone model, respectively (Fig. 4a). In the cartilage model, this difference increased to approximately 5.4 times compared to the bone model. The displacement differed greatly between the sacrum and both hip bones on the SIJ as the boundary.

During maximum displacement of the pelvis (Fig. 4b), mean values in all walking phases averaged 1.9 mm in the cartilage model and 1.1 mm in the bone model, indicating a decrease in displacement to approximately 57% compared to the cartilage model throughout all walking phases.

### Equivalent stress distribution within sacroiliac joint cartilage

**Figure 5:**
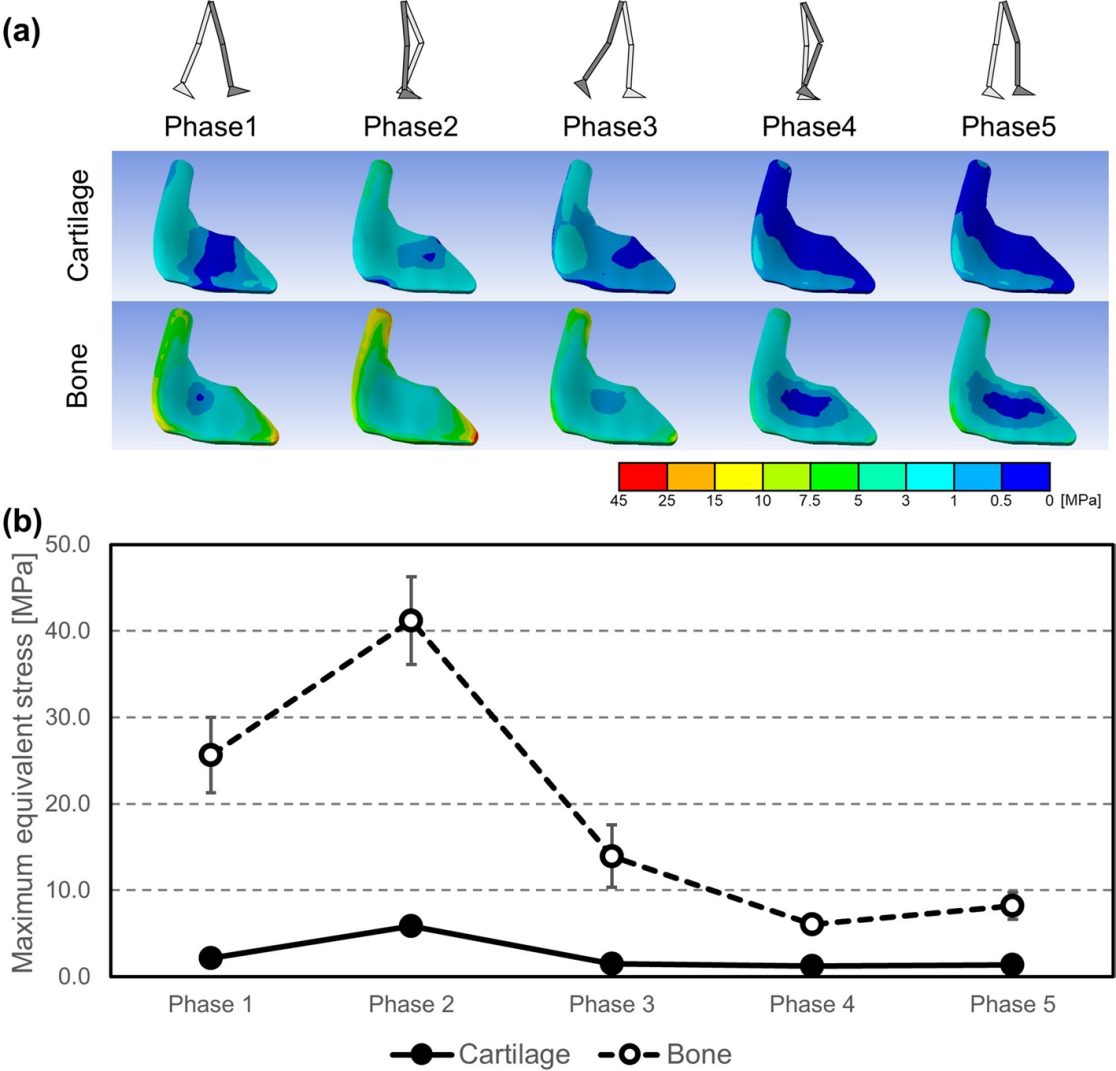
(**a**) Equivalent stress distribution of right sacroiliac joints (SIJs) on a representative example shown from left, and (**b**) change of mean values of maximum equivalent stress in right SIJs.

In the cartilage model, equivalent stresses were concentrated in the front of the SIJ cartilage throughout all walking phases and increased to approximately 5.8 MPa in the upper part of the SIJ cartilage during the stance phase (phases 1, 2, 3) (Fig. 5a). During phase 2, where only a single leg supports the whole-body weight, the equivalent stresses increased remarkably to approximately 3.8 × the extent compared to the other walking phases and averaged 5.8 MPa (Fig. 5b). In the swing phase (phases 4, 5), the equivalent stress was similar at the lower aspect of the SIJ cartilage compared to the stance phase (phases 1, 2, 3) at approximately 1.0 MPa, and maximum values decreased as a whole. Regarding the maximum equivalent stress of the right SIJ cartilage, the values at phase 2, when the maximum equivalent stress was highest, averaged 41.2 MPa in the bone model, i.e., in phase 2 the values of the bone model increased to approximately 700% of the cartilage model.

### Loads of the pelvic ligaments

**Figure 6:**
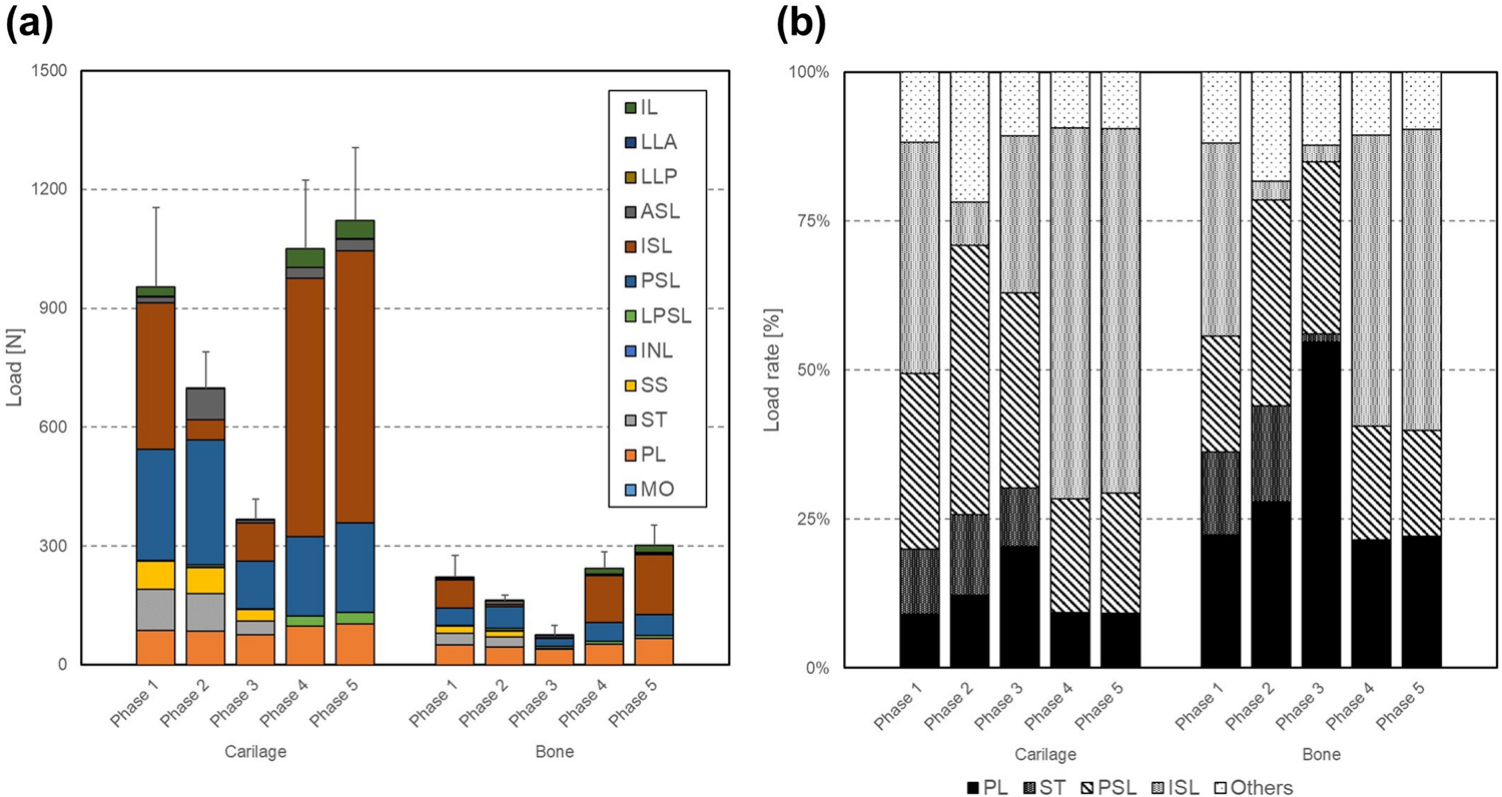
Comparison of (**a**) the mean loads and (**b**) the mean load rates on ligaments of the right part of the pelvis between the bone and cartilage model. The loads of ligaments are the sum of each ligament, which has 2–30 spring components. Here, the loads on pubic ligament PL, anterior longitudinal ligament LLA, and posterior longitudinal ligament LLP were halved due to their location at the center of the spinopelvic complex.

Though the loads varied depending on the walking phase and the ligament, the loads of the bone model decreased to approximately 23% compared to the cartilage model. In particular, the loads on the ASL, ISL and PSL as well as the SS and ST decreased to less 23%, and only the ISL and PSL decreased remarkably more than 200 N. In the cartilage model, the loads increased in the swing phase (phases 4, 5) and decreased in the stance phase (phase 1, 2, 3) (Fig. 6a). The loading rate on PSL and ISL accounted for 81% (maximum PSL 45%, ISL 62%), and was much higher than others throughout all walking phases. In addition, only on the stance phase (phases 1, 2, 3) the ST was loaded and its load rate was approximately 11% (Fig. 6b).

### Deformation of sacroiliac joint

**Figure 7:**
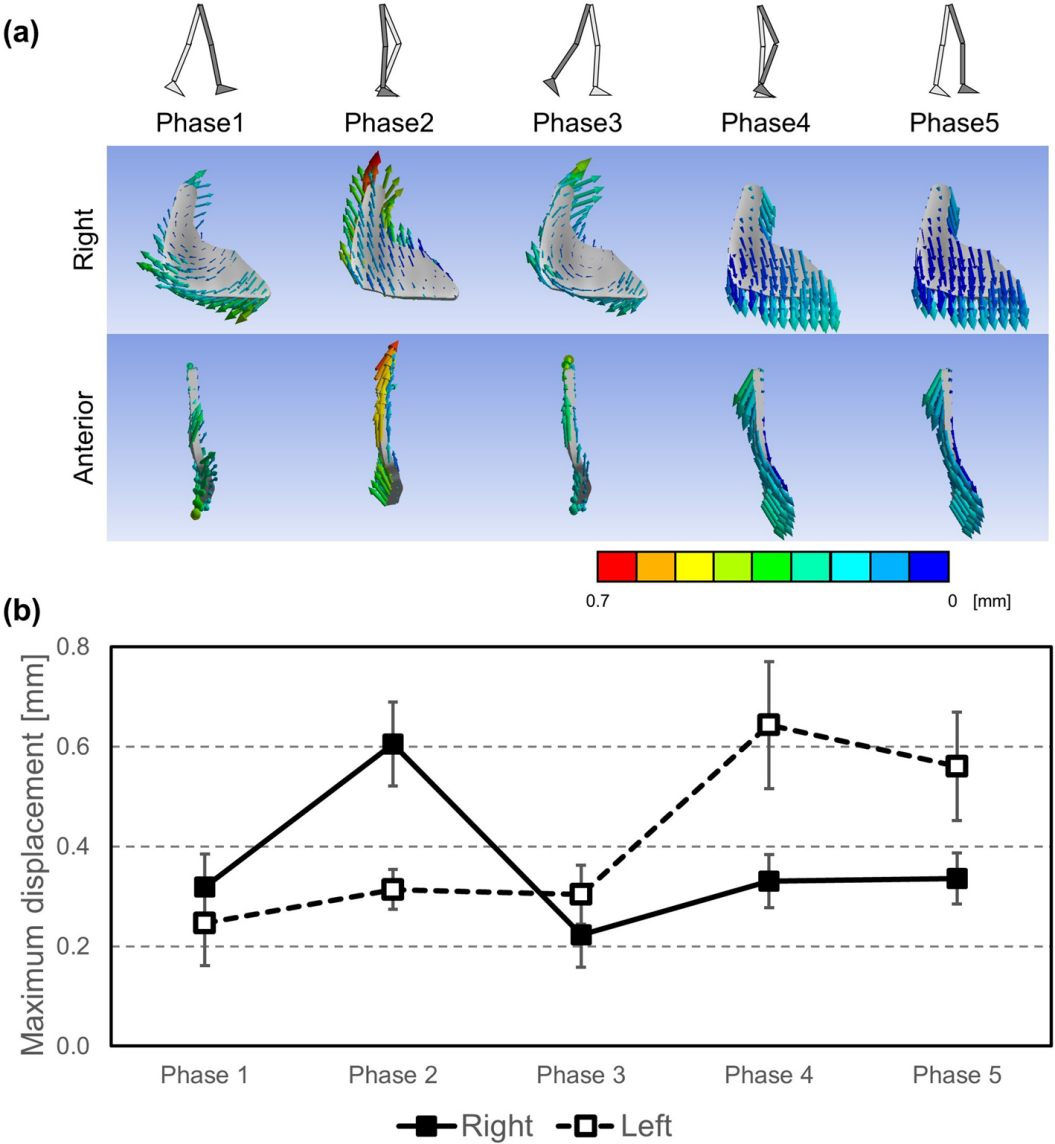
(**a**) Resultant displacement vector diagrams of sacroiliac joints (SIJs) with the cartilage model on a representative example. Right (1st line) indicates diagrams on right SIJs shown from left, and anterior (2nd line) indicates diagrams on right SIJs shown from anterior. (**b**) Change of mean values of maximum resultant displacement of right and left SIJs.

During the stance phase (phases 1, 2, 3), the SIJ cartilage was mainly deformed cranially centered on the lower part of the SIJ cartilage and the ilium went away from the sacrum on the same part. Meanwhile, during the swing phase (phases 4, 5), the SIJ cartilage was mainly deformed caudally centered on the upper part of the SIJ cartilage and the ilium went away from the sacrum on the same part (Fig. 7a). The maximum displacement of the SIJ cartilage was approximately 0.3 mm and 0.6 mm during the swing and stance phase (phases 1, 2, 3), respectively (Fig. 7b).

### Nutation and counter-nutation of the sacroiliac joint

**Figure 8:**
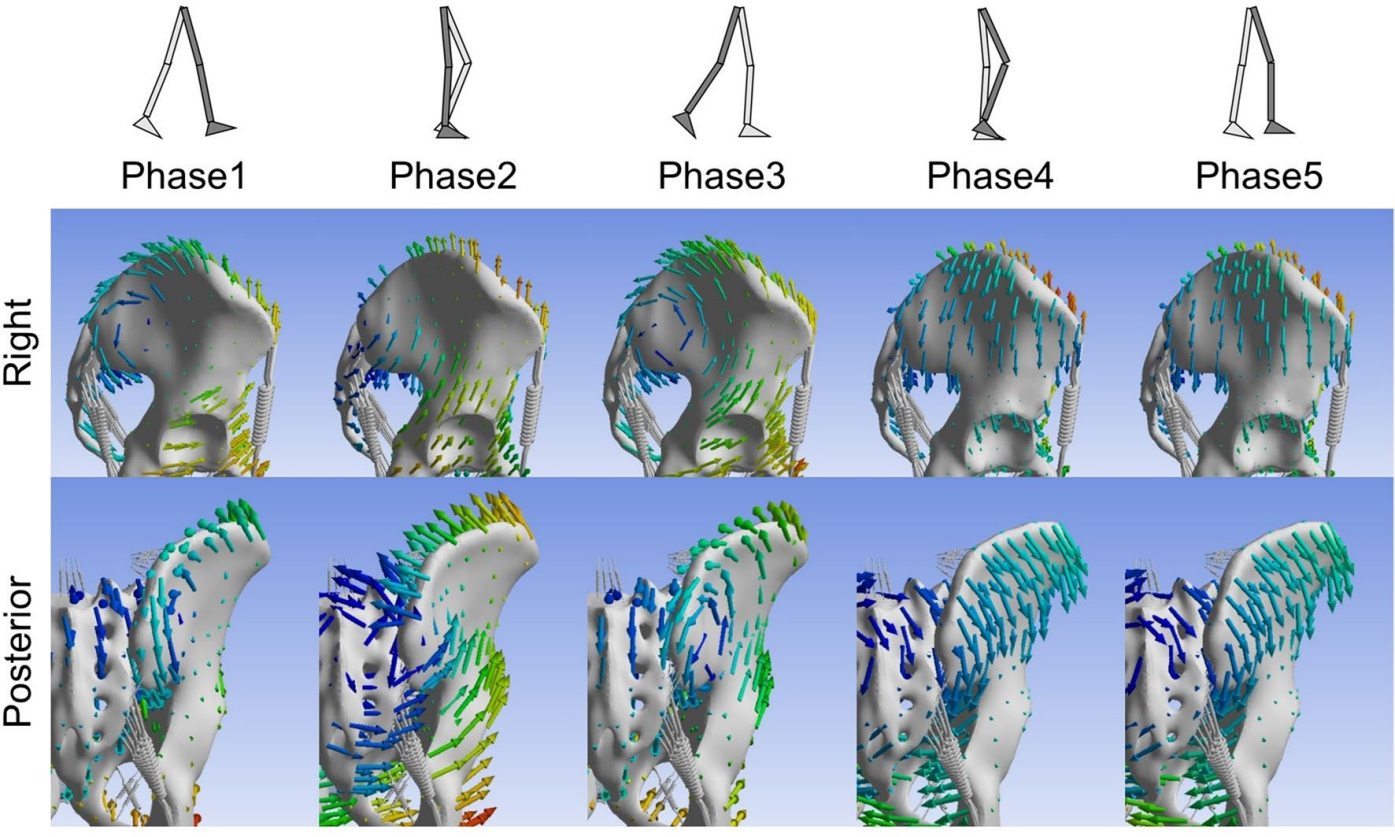
Resultant displacement vector diagrams of pelvis on a representative example. Right (1st line) and posterior (2nd line) indicate diagrams shown from right and posterior, respectively.

During the stance phase (phases 1, 2, 3), the ilium was elevated relative to the sacrum, and the sacrum had a nutation movement relative to the ilium. Meanwhile, during the swing phase (phases 4, 5), the ilium was lowered relative to the sacrum, and the sacrum moved in a counter-nutation sense relative to the ilium (Fig. 8).

## Discussion

### The sacroiliac joint appears to have a major effect on pelvic loading especially under walking conditions

In the bone model, the difference in displacement between the ilium and sacrum was minute (0.04 mm), resulting in a continuous displacement between these bones. In this model, as a result, the equivalent stress of the SIJ cartilage defined at the cortical bone was comparably high. This equivalent stress indicates load concentrations at the bony transition, to the end that in a “fused condition” premature failure of the adjacent bone may occur.

In the cartilage model including the SIJ, the difference in displacement between the ilium and sacrum was 0.23 mm, which seemed high when compared to the bone model. Loading across the bones seemed discontinuous, with the SIJ acting as a damper. These results were in line with the work of the group of Takayama^5^. Therefore, the role of the SIJ as a shock absorber can be confirmed based on the findings presented here. Potentially, the fat situated within the interosseous ligaments plays an important role in shock absorption^3,17^. Owing to this, the pelvis obtains additional elasticity. The ligaments surrounding the posterior do work additionally to help distribute the loads from the upper to lower body parts by means of increased areas of force transmission, but may at the same time be the potential failure site in case of injury^18^. These findings suggest that the SIJs forming part of the pelvis help dissipate loads and therefore be vital for non-traumatic loading at the spine-leg transition. The SIJ was found to be an area where stress peaked on the pelvic ring from the bone model. It is therefore speculated that it may be important for the structure of the pelvic ring to have a joint structure that relieves stress concentration at this area.

The load rates at the PSL and ISL were approximately 70% throughout all walking phases and much higher than in other areas. In phase 2 of the gait cycle when only a single leg supports the whole-body weight and when the equivalent stress of the SIJ cartilage peaked, their load rate decreased to approximately 52%. On the other word, when the stress of the SIJ cartilage increases, the loads of the pelvic ligaments decrease. This mechanical finding is in line with the morphology of the SIJ which is composed of an anterior synovial joint region and a posterior ligamentous region^3,19^. In particular, it was considered that the anterior synovial joint region helps carry the compression load from an upper body weight, and the ligamentous region the tensile load from the lower limb in a simplified model.

It can be assumed that in the humans walking upright and bipedal, the SIJ has become more resilient to support the weight of the upper body even under relatively unfavourable levers which constantly change when walking^6^. Cohen proposed that the SIJ is designed primarily for stability^1^. However, if the SIJ is too tightly connected for stability, this may result in a biased load with peak effects. Like the current SIJ, although supported by tough ligaments, the SIJ was considered to need to have some mobility.

Surgical SIJ fixation is a treatment that minimizes SIJ motion^6,20^, and there are fixation methods aiming the ossification on the articular surface following surgery^21,22^. These groups’ experiments have been performed with cadavers^23^ or FEM^24–26^ in order to assess the fixation. However, these only indicate a condition immediately after the surgery. In the medium to long term time frame, the actual post-fixation state may be close to the bone model presented here. The SIJ fixations suppress the SIJ deformation and relieve pain, but it could be assumed that stress of the SIJ cartilage increased due to the ossification, which may further promote bone formation and damage the SIJ when a sudden or unexpected external force is applied.

### Movements of sacroiliac joint differ under walking conditions compared to static loading

On this finite element analysis, the pelvis of the free leg moved relatively inferiorly when walking. This corresponds to reports stating that the pelvis is depressed to a swing leg on walking^27^. In addition, the SIJ had small motion of less than 1 mm, and moved separately on the left and right SIJ. The range of the SIJ movements has been investigated with various methods; on patients with SIJ disorders 0.7 mm by Sturesson et al.^4^, 0.7 mm by Jacob and Kissling^28^, and 0.3 mm by Sturesson et al.^29^, on healthy individuals 0.47 mm by Kibsgård et al.^30^, on cadavers 0.8 mm by Miller et al.^31^, less than 1 mm by Takayama^5^ and 0.3 mm by Hammer et al.^11^, and on FEM 0.3 mm by Bruna-Rosso et al.^24^. Our study showed that displacement of SIJ cartilage was ranged from 0.3 to 0.6 mm, which was similar to previous studies. It was previously demonstrated that most of the sacral movement takes place around a transverse axis, situated at the level of the second sacral vertebra. Iliac rotation relative to the sacrum was later named nutation and counter-nutation^19^, thought ambiguity exists as nutation was also named the combined rotation and translation movement of the sacrum relative to both innominate bones^27^. The above studies^4,11,28–30^ have reported that the sacrum rotates approximately 2° on the sagittal plane, which seems to indicate such movement. Bruna-Rosso et al. have shown the rotation of the sacrum with vector diagrams in FEM^24^. In this study, the relative nutation and counter-nutation movements of the sacrum during bipedal walking were visualized, which had not been clarified so far. On the side of the stance leg, the sacrum moved into the nutation position. Meanwhile, on the side of the free leg, the sacrum moved into the counter-nutation position. The nutation is a movement in which the sacrum rotates forward when the load from an upper body is applied on the upper of the sacrum and pushes down the promontory. At this time, the ground reaction force is applied via the femora to both hipbones, and the ilium tilts backwards. This promotes the nutation. However, the ligaments restrict the movements in order to avoid too ample the nutation^27^. This nutation mechanism is based on both legs standing and cannot be fully replicated when walking. From these results, the nutation mechanism during walking was interpreted as follows: On the standing leg, the nutation was facilitated by the load from an upper body and the ground reaction force as in the case of double-leg standing. Meanwhile, in the swing leg, this movement was not facilitated, and the sacrum performed a counter-nutation, as the ilium was pulled down by the weight of the lower limb. It is impossible that the left and right SIJs perform individual nutation or counter-nutation movements if the pelvis is only modelled as one bone. Given the left and right SIJ showed separate movements, it was possible to walk on a human bipedal walking with a standing leg and a free leg alternatively. This suggests that the SIJs plays an important role in the human bipedal walking mechanism, but very little information is to date available on the nutation and counter-nutation during walking, and these findings need to be substantiated further.

## Conclusions

This simulation was performed for the first time by implementing actual 3D walking data into a finite element model of the pelvis elaborated from large-scale anatomical studies. As a result, the mechanical state of SIJs during bipedal walking was visualized, and the movements of the SIJ and the loads of the SIJ cartilage and surrounding ligaments could be quantified. The walking loads caused distortion of the whole pelvis and the stress concentrated at the SIJ due of the morphology of the pelvic ring. Modeling the SIJ into the pelvic ring, stress concentrations was received, and surrounding ligaments carried the loads. It was found that the SIJ had the shock absorbing mechanism during walking. For the first time, this study, the extent of motion of the SIJ and the relative nutation and counter-nutation movements of the sacrum during bipedal walking were visualized. This novel information provides a scientifically informed basis for clinical discussions regarding the state of the SIJ dysfunction caused from the timing of pain induction during walking, gait changes, and surgical intervention.
